# Supplementary material for: Heterochromatic gene silencing controls CD4+ T cell susceptibility to regulatory T cell-mediated suppression in a murine allograft model
Source: Nat Commun. 2025 Jan 10;16:566. doi: 10.1038/s41467-025-55848-4 (PMC11723947; doi:10.1038/s41467-025-55848-4)
Supplement: Supplementary file 1 — Supplementary Information [file 41467_2025_55848_MOESM1_ESM.pdf]

SUPPLEMENTARY INFORMATION FILE

Heterochromatic gene silencing controls CD4$^+$ T cell susceptibility to regulatory T cell-mediated suppression in a murine allograft model

Julie Noguerol[1], Karl Laviolette[1#], Margot Zahm[1#], Adeline Chaubet[1], Ambrine Sahal[2], Claire Détraves[1], Romain Torres[1], Clothilde Demont[1], Véronique Adoue[1], Carine Joffre[2], Florence Cammas[3,4,5], Joost PM van Meerwijk[1], Olivier P Joffre[1✉]

[1] Infinity, Toulouse Institute for Infectious and Inflammatory Diseases, University of Toulouse, Inserm U1291, CNRS U5051, Toulouse, France.

[2] Centre de Recherche en Cancérologie de Toulouse, Université de Toulouse, Inserm U1037, CNRS U5071, Toulouse, France.

[3] Institut de Recherche en Cancérologie de Montpellier, INSERM U1194, Université Montpellier, 34298 Montpellier, France.

[4] Institut Régional du Cancer Montpellier, Université Montpellier, 34298 Montpellier, France

[5] Present address: Institute of Human Genetics, CNRS UMR9002 University of Montpellier, 34396 Montpellier, France.

[#]These authors contributed equally to this work.

[✉]**Corresponding author:**

olivier.joffre@inserm.fr

CONTENT

Supplementary Figure 1. No detectable alteration of the T cell compartment in HP1α-deficient mice. Related to Figure 1.

Supplementary Figure 2. HP1α-deficiency does not impact on Th1 or Th17 priming *in vitro*. Related to Figure 2.

Supplementary Figure 3. HP1α-deficiency unlocks Th1 and Th17 gene expression programs. Related to Figure 3.

Supplementary Figure 4. No detectable alteration of the T cell compartment in HP1γ-deficient mice. Related to Figure 4.

Supplementary Figure 5. HP1γ-deficiency does not impact on Th1, Th17 or Treg priming *in vitro*. Related to Figure 5.

Main Flow Cytometry gating strategies.
Gating strategy used to analyze BM allograft rejection (Figures 1, 4 and S4)
Gating strategy used to analyze TCR Vb6+ Tconv (Figures 2 and 5)
Gating strategy used to analyze human Tconv (Figure 7)

Uncropped western-blots. Uncropped western-blot of figures S1B and S4C.

Supplementary Figure 1. No detectable alteration of the T cell compartment in HP1α-deficient mice. Related to Figure 1.

(a) Gene expression levels in naive $CD4^+$ T cells. Data are means ± SEM of three independent experiments. (b, c) HP1s expression levels in naive $CD4^+$ T cells. Representative western-blots (b) and normalized average expression levels (c) are shown. Data are means ± SEM of four independent experiments. Statistical significance was calculated using unpaired, two-tailed t test. (d) Representative dot-plots showing CD4 and CD8 expression by thymocytes. (e) Relative proportions of $CD4^-CD8^-$ (double-negative, DN), $CD4^+CD8^+$ (double-positive, DP), $CD4^+CD8^-$ (CD4 single-positive, CD4 SP) and $CD4^-CD8^+$ (CD8 single-positive, CD8 SP) thymocytes. (f) Percentage of Treg among CD4 SP. (g-h) Percentage of CD4 and CD8 T cells in the spleen (g) and mesenteric lymph nodes (mLN) (h). (i) Percentage of Treg. Data are means ± SEM of two (f) or three (e, g-i) independent experiments. (j) Representative dot-plots showing CD44 and CD62L expression by spleen $CD4^+$ T cells. (k) Relative proportions of Naive and Effector-Memory (e-m) cells among spleen $CD4^+$ T cells. Data are means ± SEM of four independent experiments. (l) Representative dot-plots showing CD44 and CD62L expression by spleen $CD8^+$ T cells. (m) Relative proportions of Naive, Effector-Memory (E-M) and Central Memory (C-M) cells among spleen $CD8^+$ T cells. Data are means ± SEM of four independent experiments. (n) Percentages of cytokine-producing memory $CD4^+$ T cells. Data are means ± SEM of four independent experiments. (o-p) Volcano plots showing results of differential gene expression analyses between WT and HP1α KO memory (o) or naive (p) spleen $CD4^+$ T cells. Red and black dots represent genes with higher expression in HP1α KO and WT cells, respectively. Gray dots represent genes that failed to reach the FDR threshold of 0.05 and the absolute log2 fold change threshold of 1. (q) Percentage of $CD4^+CD25^-CD62^{low}CD44^{high}$ cells within naive Tconv. Data are means ± SEM of five independent experiments. (r) Percentage of $CD4^+Foxp3^+Thy1.1^+$ cells within Treg. Individual values and the mean of seven independent experiments are shown. Source data are provided in the Source data file.

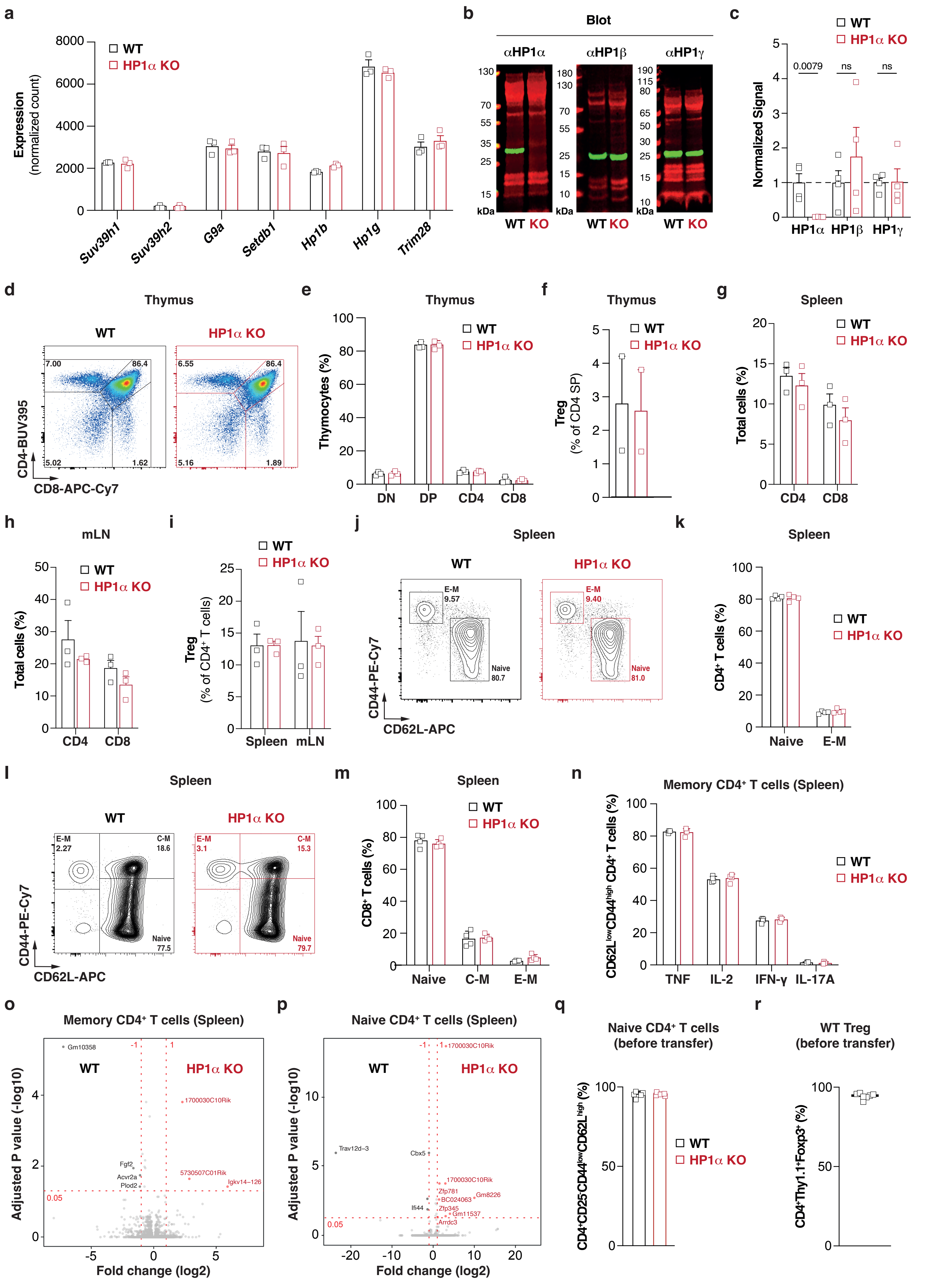
a
Expression
(normalized count)
8000
6000
4000
2000
0
WT
HP1α KO
Suv39h1
Suv39h2
G9a
Setdb1
Hp1b
Hp1g
Trim28
b
Blot
αHP1α
αHP1β
αHP1γ
130
70
55
35
25
15
kDa
180
130
70
55
25
15
10
kDa
190
115
80
65
50
30
25
15
10
kDa
WT KO
WT KO
WT KO
c
WT
HP1α KO
Normalized Signal
0.0079
ns
ns
HP1α
HP1β
HP1γ
d
Thymus
WT
HP1α KO
7.00
86.4
5.02
1.62
6.55
86.4
5.16
1.89
CD4-BUV395
CD8-APC-Cy7
e
Thymus
Thymocytes (%)
DN
DP
CD4
CD8
f
Thymus
Treg
(% of CD4 SP)
g
Spleen
Total cells (%)
CD4
CD8
h
mLN
Total cells (%)
CD4
CD8
i
Treg
(% of CD4+ T cells)
Spleen
mLN
j
Spleen
WT
HP1α KO
E-M
9.57
Naive
80.7
E-M
9.40
Naive
81.0
CD44-PE-Cy7
CD62L-APC
k
Spleen
CD4+ T cells (%)
Naive
E-M
l
Spleen
WT
HP1α KO
E-M
2.27
C-M
18.6
Naive
77.5
E-M
3.1
C-M
15.3
Naive
79.7
CD44-PE-Cy7
CD62L-APC
m
Spleen
CD8+ T cells (%)
Naive
C-M
E-M
n
Memory CD4+ T cells (Spleen)
CD62LlowCD44high CD4+ T cells (%)
TNF
IL-2
IFN-γ
IL-17A
o
Memory CD4+ T cells (Spleen)
Gm10358
WT
HP1α KO
1700030C10Rik
Fgf2
Acvr2a
Plod2
5730507C01Rik
Igkv14–126
0.05
Adjusted P value (-log10)
Fold change (log2)
p
Naive CD4+ T cells (Spleen)
1700030C10Rik
Trav12d–3
Cbx5
Zfp781
BC024063
Gm8226
Ifi44
Zfp345
Gm11537
Arrdc3
0.05
Adjusted P value (-log10)
Fold change (log2)
q
Naive CD4+ T cells
(before transfer)
CD4+CD25-CD44lowCD62Lhigh (%)
WT
HP1α KO
r
WT Treg
(before transfer)
CD4+Thy1.1+Foxp3+ (%)

Supplementary Figure 2. HP1α-deficiency does not impact on Th1 or Th17 priming *in vitro*. Related to Figure 2.

(a) Representative dot-plots showing CD25 and CD69 expression by naive $CD4^+$ T cells previously activated, or not, for two days. (b) Percentage of $CD4^+$ T cells coexpressing CD69 and CD25 before (D0) or after (D2) *in vitro* activation. Data are means ± SEM of seven biological replicates from four independent experiments. (c-k) WT and HP1α KO naive $CD4^+$ T cells were labelled with CellTrace Violet (CTV) and cultured in Th1-polarizing conditions. (c) Representative histograms showing CTV dilution after four days of culture. (d-e) Proliferation index (d) and percentage of divided cells (e) as calculated from the data in (c). Data are means ± SEM of four independent experiments. (f) Representative dot-plots showing IFN-γ and IL-17A production by Th1 cells. (g) Percentage of T cells producing IFN-γ after 3 and 6 days of culture. Data are means ± SEM of four (day 3) or five (day 6) independent experiments. (h) Representative histograms showing Tbet expression by Th1 cells. (i) Average Tbet expression per Th1 cell (Geomean). Data are means ± SEM of four independent experiments. (j) Percentage of IFN-γ-producing $CD4^+$ T cells after 6 days of culture in Th1 medium containing increasing concentrations of IL-12. Data are from one experiment and are representative of two independent experiments. (k-o) WT and HP1α KO naive $CD4^+$ T cells were cultured in Th17-polarizing conditions. (k) Representative dot-plots showing IL-17A and GM-CSF production of by T cells after 6 days of culture. (l, m) Percentage of IL-17A (l) or GM-CSF (m) producers among T cells after 3 and 6 days of culture. Data are means ± SEM of three (Day 3) or five (Day 6) independent experiments. (n) Representative histograms showing RORγt expression by T cells after 6 days of culture. (o) Average RORγt expression per Th17 cell (Geomean). Data are means ± SEM of four independent experiments. Source data are provided in the Source data file.

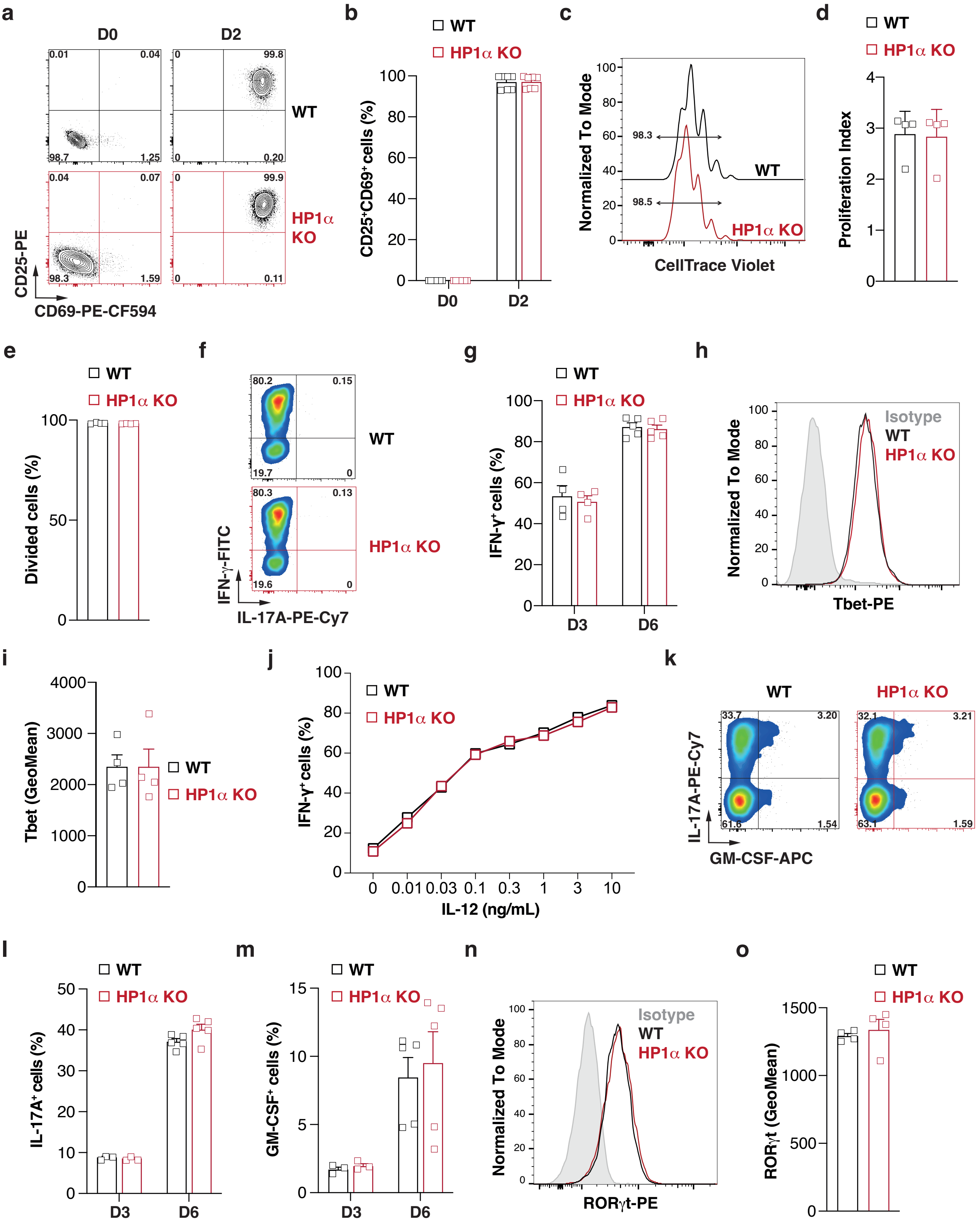
a
D0
D2
0.01
0.04
98.7
1.25
0
99.8
0
0.20
WT
0.04
0.07
98.3
1.59
0
99.9
0
0.11
HP1α KO
CD25-PE
CD69-PE-CF594
b
WT
HP1α KO
CD25+CD69+ cells (%)
D0
D2
c
Normalized To Mode
98.3
98.5
WT
HP1α KO
CellTrace Violet
d
Proliferation Index
e
Divided cells (%)
f
80.2
0.15
19.7
0
80.3
0.13
19.6
0
IFN-γ-FITC
IL-17A-PE-Cy7
g
IFN-γ+ cells (%)
D3
D6
h
Isotype
Tbet-PE
i
Tbet (GeoMean)
j
IL-12 (ng/mL)
k
33.7
3.20
61.6
1.54
32.1
3.21
63.1
1.59
GM-CSF-APC
l
IL-17A+ cells (%)
m
GM-CSF+ cells (%)
n
RORγt-PE
o
RORγt (GeoMean)

Supplementary Figure 3. HP1α-deficiency unlocks Th1 and Th17 gene expression programs. Related to Figure 3.

(a) GO enrichment analyses of genes more highly expressed in Tconv HP1α KO than WT. (b) GSEA of KEGG pathways mmu04657 (IL-17A signaling pathways), mmu05321 (Inflammatory bowel disease) and mmu04064 (NF-kappa B signaling pathway) performed using transcriptomes of HP1α KO and WT Tconv. (c) GO enrichment analyses of genes more highly expressed in HP1α KO Tconv than in WT Tconv exposed to Treg. (d) KEGG enrichment analyses of genes more highly expressed in HP1α KO Tconv than in WT Tconv exposed to Treg and associated with peaks more open in HP1α KO Tconv than in WT Tconv exposed to Treg. (e) Venn diagram showing the relationship between the genes more highly expressed in Treg-exposed WT Tconv than in HP1α KO Tconv and the genes associated with peaks more open in WT Tconv than in HP1α KO Tconv exposed to Treg. (f) Expression levels of the 78 genes identified in (e). The horizontal bar represents the median, and the top and bottom of the box the upper and lower quartile, respectively. The whiskers go from the minimum to the lower quartile and from the upper quartile to the maximum. Statistical significance was calculated using the Pairwise Wilcoxon Rank Sum Test (two-tailed). (g) GO enrichment analyses of the 78 genes identified in (e). Source data are provided in the Source data file.

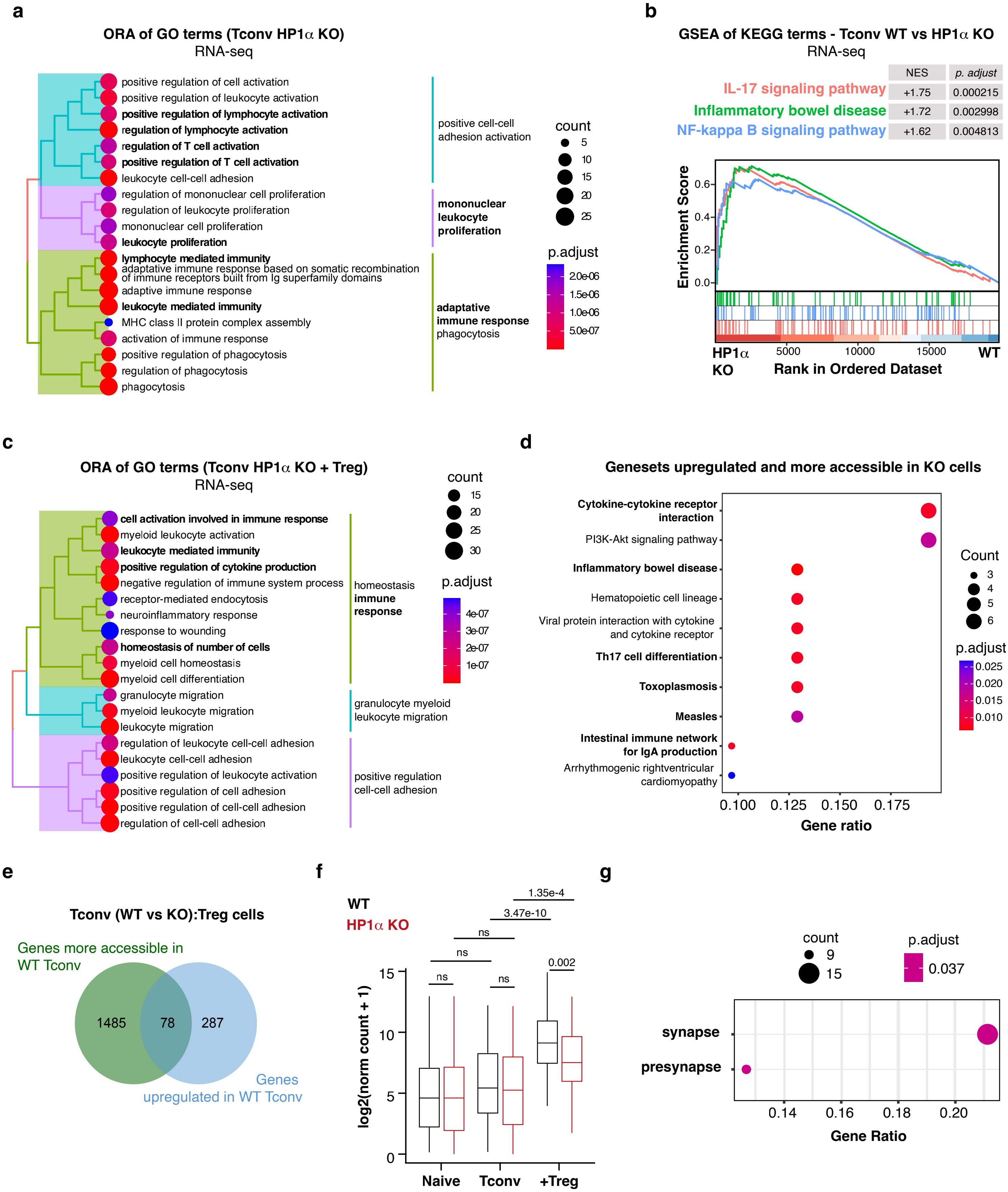

a
ORA of GO terms (Tconv HP1α KO)
RNA-seq
positive regulation of cell activation
positive regulation of leukocyte activation
positive regulation of lymphocyte activation
regulation of lymphocyte activation
regulation of T cell activation
positive regulation of T cell activation
leukocyte cell-cell adhesion
regulation of mononuclear cell proliferation
regulation of leukocyte proliferation
mononuclear cell proliferation
leukocyte proliferation
lymphocyte mediated immunity
adaptative immune response based on somatic recombination of immune receptors built from Ig superfamily domains
adaptive immune response
leukocyte mediated immunity
MHC class II protein complex assembly
activation of immune response
positive regulation of phagocytosis
regulation of phagocytosis
phagocytosis
positive cell-cell adhesion activation
mononuclear leukocyte proliferation
adaptative immune response phagocytosis
count
5
10
15
20
25
p.adjust
2.0e-06
1.5e-06
1.0e-06
5.0e-07
b
GSEA of KEGG terms - Tconv WT vs HP1α KO
RNA-seq
NES
p. adjust
IL-17 signaling pathway +1.75 0.000215
Inflammatory bowel disease +1.72 0.002998
NF-kappa B signaling pathway +1.62 0.004813
Enrichment Score
0.6
0.4
0.2
0.0
HP1α KO
5000
10000
15000
WT
Rank in Ordered Dataset
c
ORA of GO terms (Tconv HP1α KO + Treg)
RNA-seq
cell activation involved in immune response
myeloid leukocyte activation
leukocyte mediated immunity
positive regulation of cytokine production
negative regulation of immune system process
receptor-mediated endocytosis
neuroinflammatory response
response to wounding
homeostasis of number of cells
myeloid cell homeostasis
myeloid cell differentiation
granulocyte migration
myeloid leukocyte migration
leukocyte migration
regulation of leukocyte cell-cell adhesion
leukocyte cell-cell adhesion
positive regulation of leukocyte activation
positive regulation of cell adhesion
positive regulation of cell-cell adhesion
regulation of cell-cell adhesion
homeostasis immune response
granulocyte myeloid leukocyte migration
positive regulation cell-cell adhesion
count
15
20
25
30
p.adjust
4e-07
3e-07
2e-07
1e-07
d
Genesets upregulated and more accessible in KO cells
Cytokine-cytokine receptor interaction
PI3K-Akt signaling pathway
Inflammatory bowel disease
Hematopoietic cell lineage
Viral protein interaction with cytokine and cytokine receptor
Th17 cell differentiation
Toxoplasmosis
Measles
Intestinal immune network for IgA production
Arrhythmogenic rightventricular cardiomyopathy
0.100
0.125
0.150
0.175
Gene ratio
Count
3
4
5
6
p.adjust
0.025
0.020
0.015
0.010
e
Tconv (WT vs KO):Treg cells
Genes more accessible in WT Tconv
1485
78
287
Genes upregulated in WT Tconv
f
WT
HP1α KO
1.35e-4
3.47e-10
ns
ns
ns
ns
0.002
log2(norm count + 1)
15
10
5
0
Naive
Tconv
+Treg
g
count
9
15
p.adjust
0.037
synapse
presynapse
0.14
0.16
0.18
0.20
Gene Ratio

Supplementary Figure 4. No detectable alteration of the T cell compartment in HP1γ-deficient mice. Related to Figure 4.

(a, b) Lethally-irradiated B6 mice were grafted with a mixture of B6 and B6D2F1 BM and co-injected, or not, with WT or HP1β-deficient naive $CD4^+$ T cells alone or in the presence of Treg. (a) Frequency of syngeneic and semi-allogeneic cells 21 days after engraftment. (b) Percentage of semi-allogeneic cells 21 days after engraftment. Data are means ± SEM of three independent experiments. (c, d) HP1s expression levels in naive $CD4^+$ T cells. Representative blots (c) and normalized average expression levels (d) are shown. Data are means ± SEM of three independent experiments. Statistical significance was calculated using unpaired, two-tailed t test. (e) Genes expression levels. Data are means ± SEM of three independent experiments. (f, g) CD4 and CD8 expression by thymocytes. Representative dot-plots (f) and proportions of the main thymocyte populations (g) are shown. (h) Percentage of Treg among CD4 SP. (i, j) Percentage of CD4 and CD8 T cells in the spleen and mLN. (k) Percentage of Treg among spleen and mLN $CD4^+$ T cells. Data are means ± SEM of two (h) or three (g, i-k) independent experiments. (l, n) CD44 and CD62L expression by spleen $CD4^+$ and $CD8^+$ T cells. (m) Relative proportions of Naive and E-M cells among spleen $CD4^+$ T cells. Data are means ± SEM of four independent experiments. (o) Relative proportions of Naive, E-M and C-M cells among spleen $CD8^+$ T cells. Data are means ± SEM of four independent experiments. (p) Percentages of cytokine-producing memory T cells. Data are means ± SEM of four independent experiments. (q-r) Volcano plots showing results of differential gene expression analyses between WT and HP1γ KO memory (q) or naive (r) spleen $CD4^+$ T cells. Red and black dots represent genes with higher expression in HP1γ KO and WT cells, respectively. Grey dots represent genes that are not differentially expressed. (s) Percentage of $CD4^+CD25^-CD62^{low}CD44^{high}$ cells in naive Tconv. Data are means ± SEM of five independent experiments. Source data are provided in the Source data file.

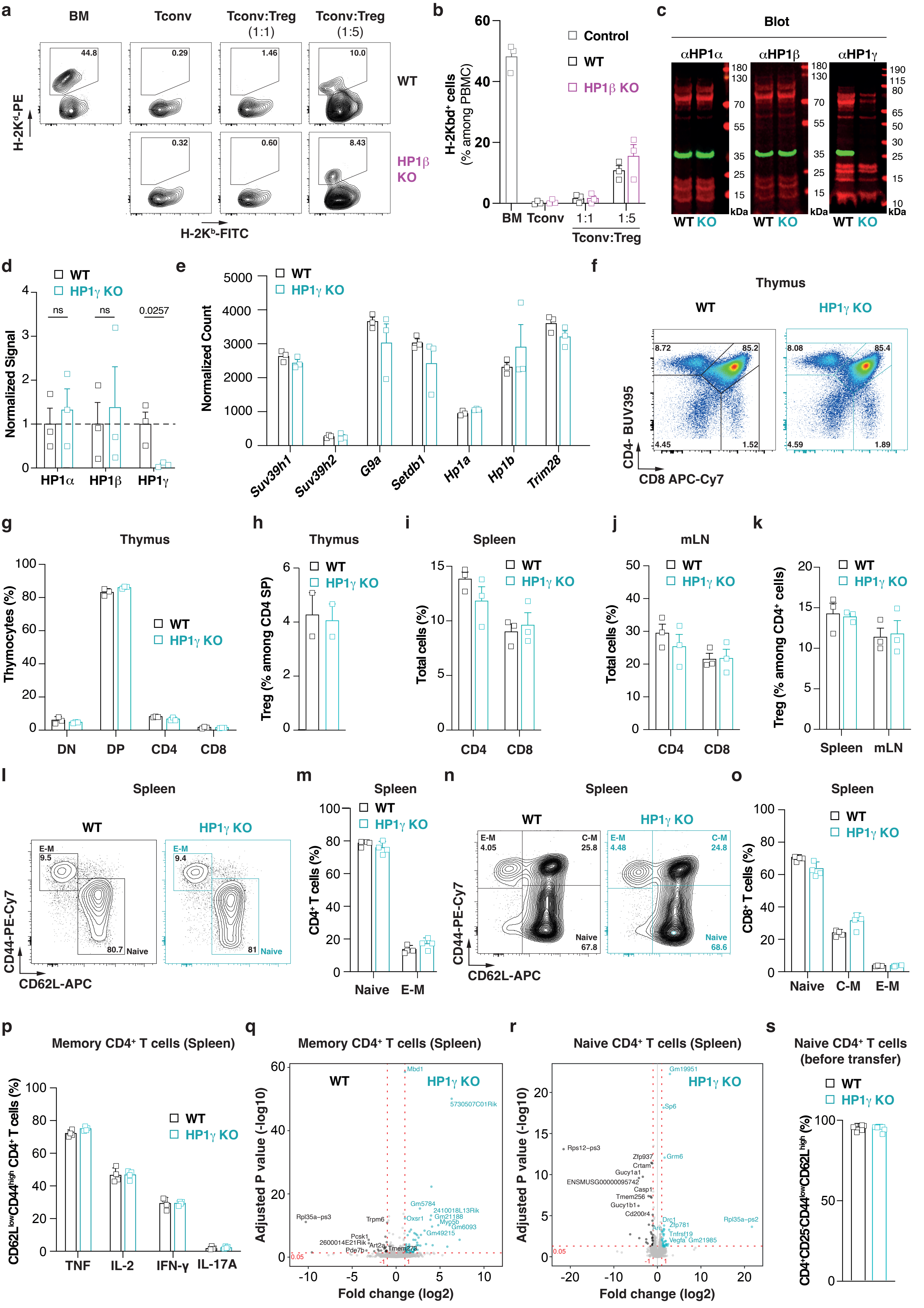

a
BM
Tconv
Tconv:Treg (1:1)
Tconv:Treg (1:5)
44.8
0.29
1.46
10.0
WT
0.32
0.60
8.43
HP1β KO
H-2K^d-PE
H-2K^b-FITC
b
Control
WT
HP1β KO
H-2Kbd+ cells (% among PBMC)
60
40
20
0
BM
Tconv
1:1
1:5
Tconv:Treg
c
Blot
αHP1α
αHP1β
αHP1γ
180
130
70
55
35
25
15
kDa
190
115
80
65
50
30
10
WT KO
d
WT
HP1γ KO
ns
ns
0.0257
Normalized Signal
HP1α
HP1β
HP1γ
e
WT
HP1γ KO
Normalized Count
5000
4000
3000
2000
1000
0
Suv39h1
Suv39h2
G9a
Setdb1
Hp1a
Hp1b
Trim28
f
Thymus
WT
HP1γ KO
8.72
85.2
4.45
1.52
8.08
85.4
4.59
1.89
CD4- BUV395
CD8 APC-Cy7
g
Thymus
WT
HP1γ KO
Thymocytes (%)
DN
DP
CD4
CD8
h
Thymus
Treg (% among CD4 SP)
i
Spleen
Total cells (%)
CD4
CD8
j
mLN
Total cells (%)
k
Treg (% among CD4+ cells)
Spleen
mLN
l
Spleen
WT
HP1γ KO
E-M
9.5
80.7
Naive
9.4
81
CD44-PE-Cy7
CD62L-APC
m
Spleen
CD4+ T cells (%)
Naive
E-M
n
Spleen
E-M 4.05
C-M 25.8
Naive 67.8
E-M 4.48
C-M 24.8
Naive 68.6
o
Spleen
CD8+ T cells (%)
Naive
C-M
E-M
p
Memory CD4+ T cells (Spleen)
CD62L^low CD44^high CD4+ T cells (%)
TNF
IL-2
IFN-γ
IL-17A
q
Memory CD4+ T cells (Spleen)
WT
HP1γ KO
Mbd1
5730507C01Rik
Gm5784
2410018L13Rik
Gm21188
Oxsr1
Myo5b
Gm6093
Gm49215
Rpl35a−ps3
Trpm6
Pcsk1
2600014E21Rik
Art2a
Pde7b
Tmem27b
Adjusted P value (-log10)
Fold change (log2)
r
Naive CD4+ T cells (Spleen)
Gm19951
Sp6
Grm6
Rps12−ps3
Zfp937
Crtam
Gucy1a1
ENSMUSG00000095742
Casp1
Tmem256
Gucy1b1
Cd200r4
Drc1
Zfp781
Tnfrsf19
Vegfa
Gm21985
Rpl35a−ps2
0.05
s
Naive CD4+ T cells (before transfer)
CD4+CD25-CD44^low CD62L^high (%)

Supplementary Figure 5. HP1γ-deficiency does not impact on Th1, Th17 or Treg priming *in vitro*. Related to Figure 5.

(a) Representative dot-plots showing CD25 and CD69 expression by naive $CD4^+$ T cells previously activated, or not, for two days. (b) Percentage of $CD4^+$ T cells coexpressing CD69 and CD25 before (D0) or after (D2) *in vitro* activation. Data are means ± SEM of four independent experiments. (c-e) WT and HP1γ KO naive $CD4^+$ T cells were cultured in Th1-polarizing conditions. (c) Representative dot-plots showing IFN-γ and IL-17A production by T cells after 6 days of culture. (d) Percentage of T cells producing IFN-γ after 3 and 6 days of culture. Data are means ± SEM of three (day 3) or five (day 6) independent experiments. (e) Average Tbet expression per Th1 cell (Geomean). Data are means ± SEM of five independent experiments. (f-i) WT and HP1γ KO naive $CD4^+$ T cells were cultured in Th17-polarizing conditions. (f) Representative dot-plots showing IL-17A and GM-CSF production by T cells after 6 days of culture. (g, h) Percentage of IL-17A (g) or GM-CSF (h) producers among T cells after 3 and 6 days of culture in Th17 medium. (i) Average RORγt expression per Th17 cell (GeoMean). (g-i) Data means ± SEM of three independent experiments. (j) CTV-labeled WT or HP1γ KO naive CD4 T cells and freshly isolated WT Treg were co-cultured at different ratios with antigen-presenting cells loaded with anti-CD3 antibody. After 4 days of culture, inhibition of T cell proliferation by Treg was measured by flow cytometry. Data are means ± SEM of four independent experiments. Statistical significance was calculated using unpaired, two-tailed Student's t test. Source data are provided in the Source data file.

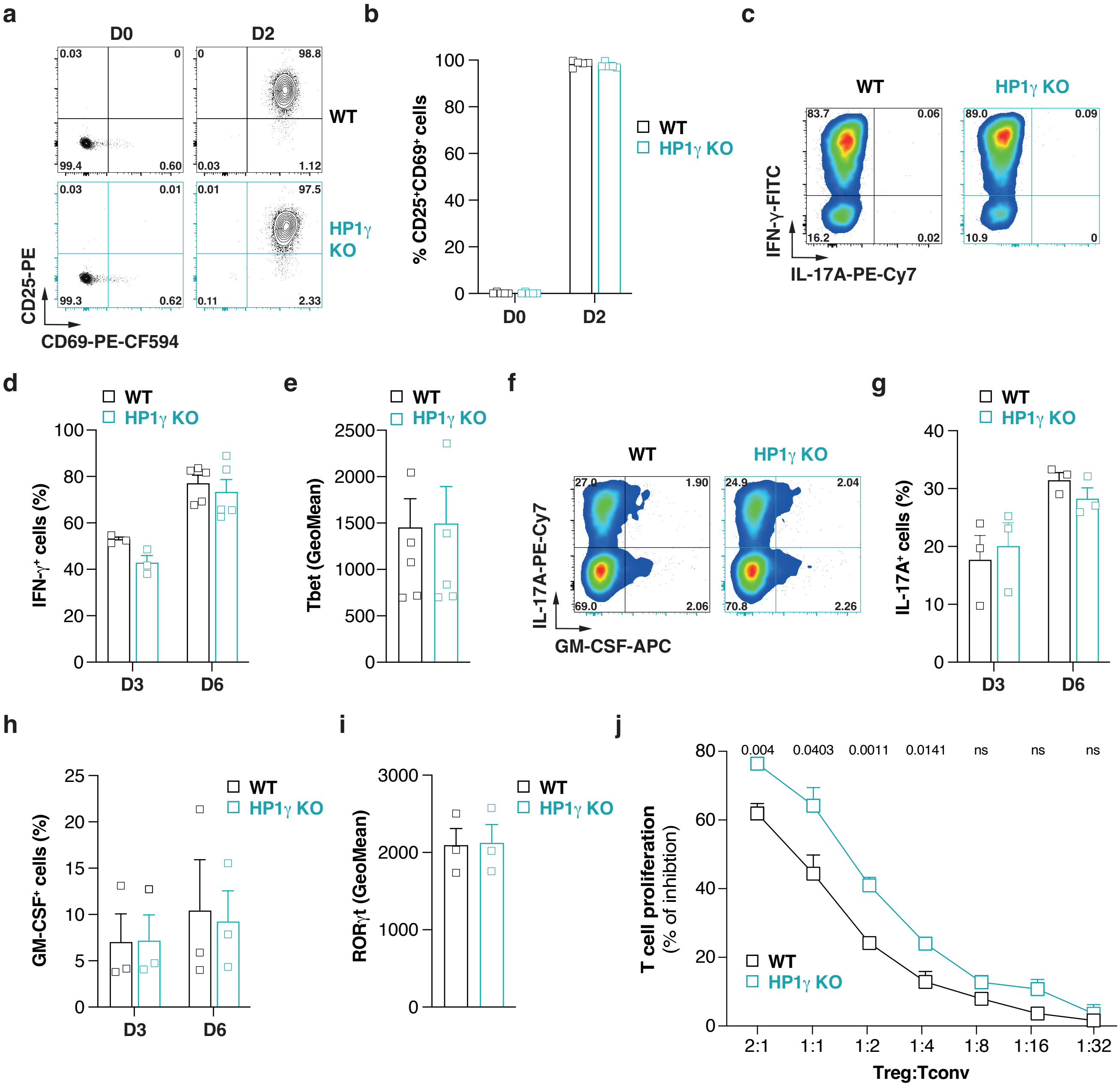

a
D0
D2
0.03
0
99.4
0.60
0
98.8
0.03
1.12
WT
0.03
0.01
99.3
0.62
0.01
97.5
0.11
2.33
HP1γ KO
CD25-PE
CD69-PE-CF594
b
% CD25+CD69+ cells
100
80
60
40
20
0
D0
D2
WT
HP1γ KO
c
WT
HP1γ KO
83.7
0.06
16.2
0.02
89.0
0.09
10.9
0
IFN-γ-FITC
IL-17A-PE-Cy7
d
WT
HP1γ KO
IFN-γ+ cells (%)
100
80
60
40
20
0
D3
D6
e
WT
HP1γ KO
Tbet (GeoMean)
2500
2000
1500
1000
500
0
f
WT
HP1γ KO
27.0
1.90
69.0
2.06
24.9
2.04
70.8
2.26
IL-17A-PE-Cy7
GM-CSF-APC
g
WT
HP1γ KO
IL-17A+ cells (%)
40
30
20
10
0
D3
D6
h
GM-CSF+ cells (%)
25
20
15
10
5
0
D3
D6
WT
HP1γ KO
i
RORγt (GeoMean)
3000
2000
1000
0
WT
HP1γ KO
j
T cell proliferation
(% of inhibtion)
80
60
40
20
0
0.004
0.0403
0.0011
0.0141
ns
ns
ns
WT
HP1γ KO
2:1
1:1
1:2
1:4
1:8
1:16
1:32
Treg:Tconv

Gating strategy used to analyze BM allograft rejection (Figures 1, 4, S4)

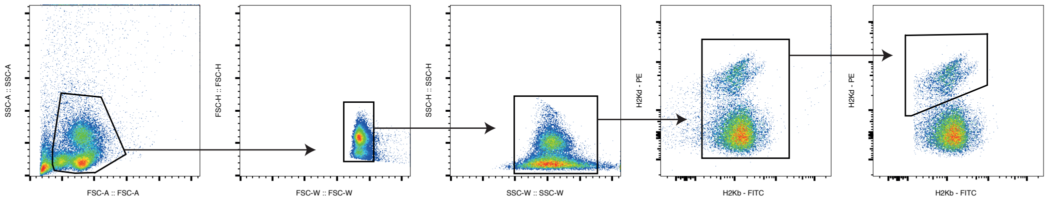

Gating strategy used to analyze TCR Vβ6⁺ Tconv (Figures 2, 5)

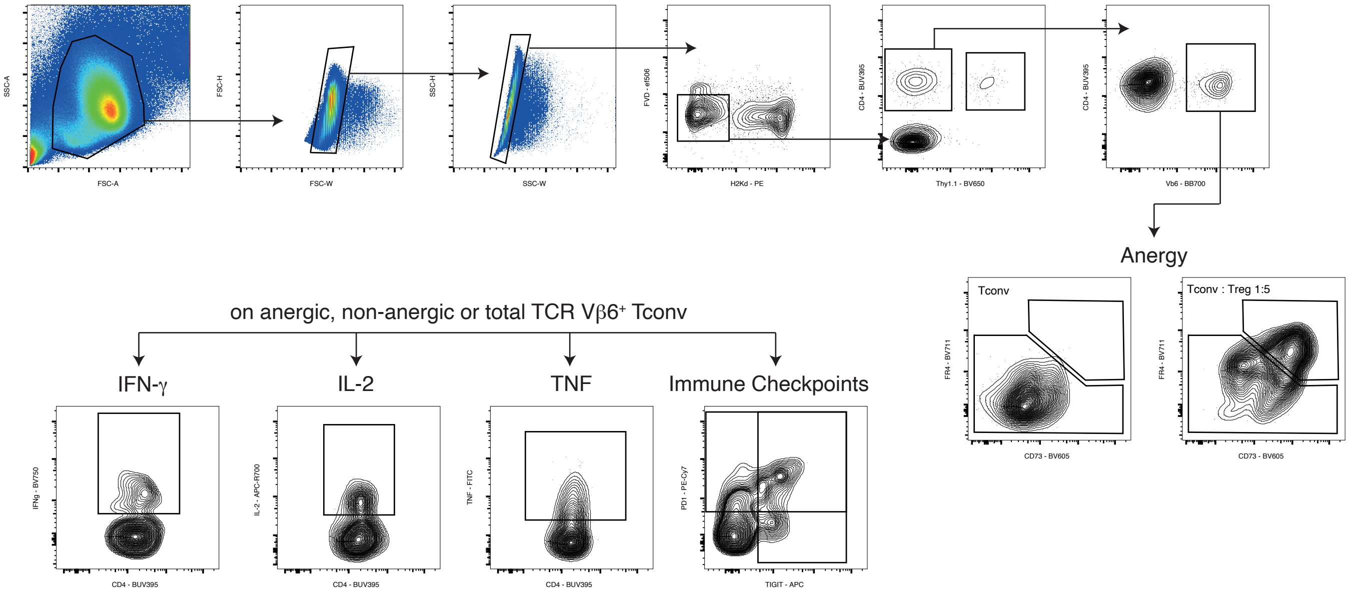

Gating strategy used to analyze human Tconv (Figure 7)

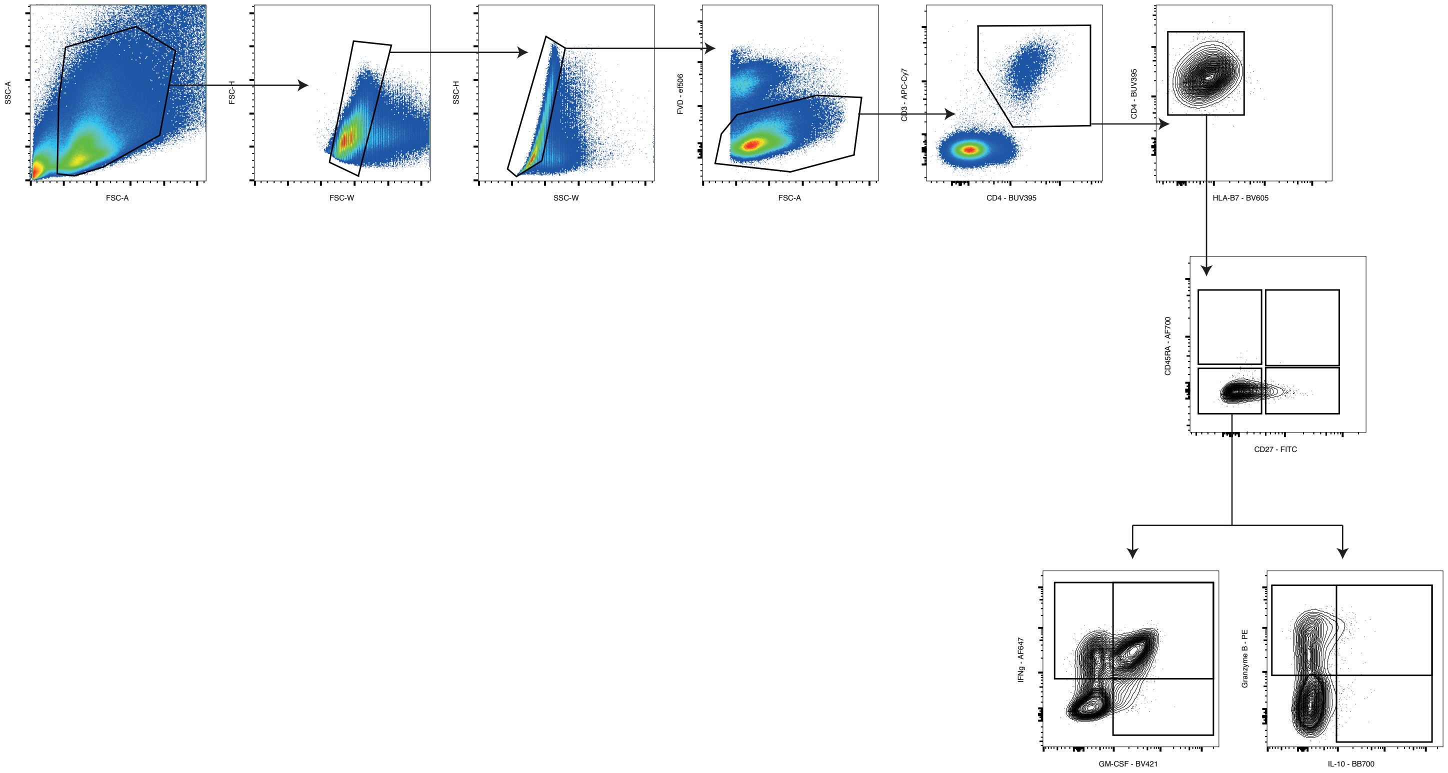

Uncropped blots of panel S1B

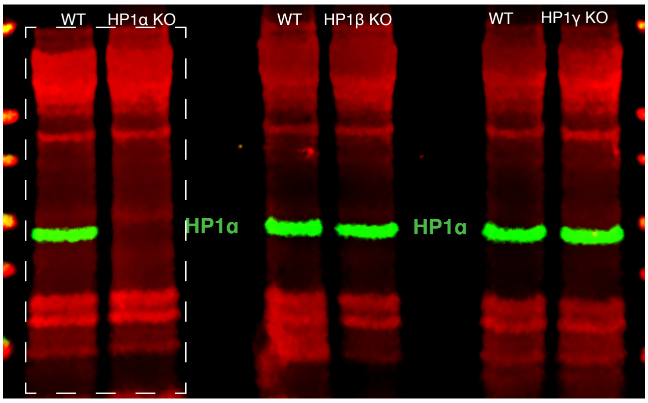

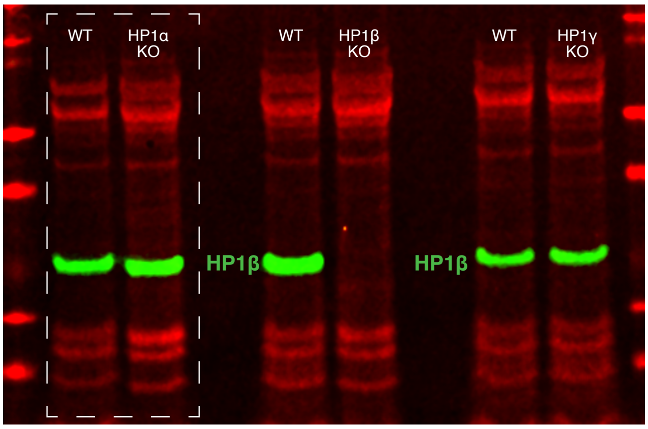

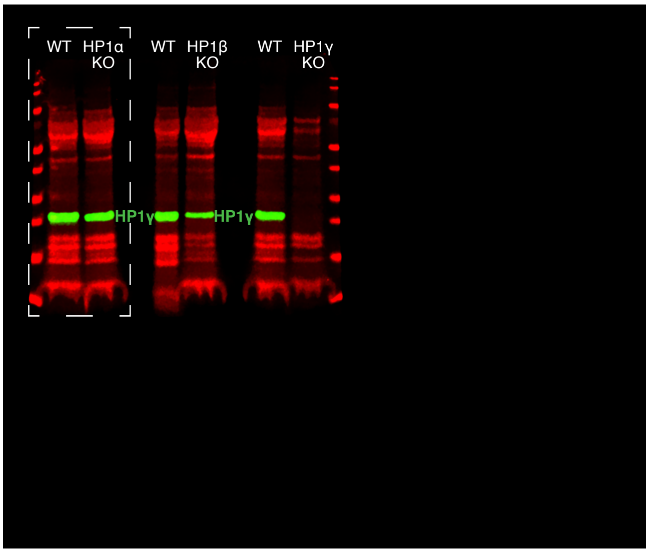

Uncropped blots of panel S4C

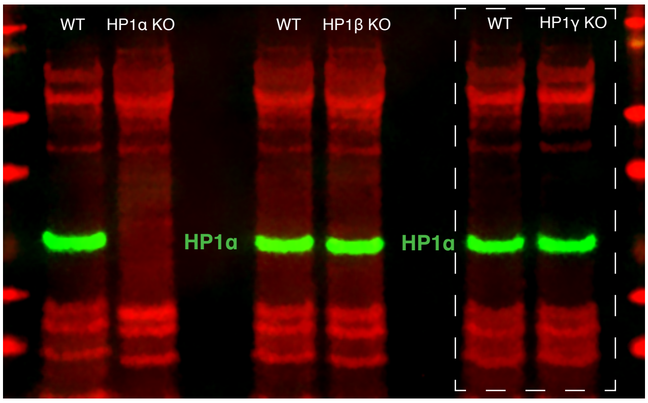

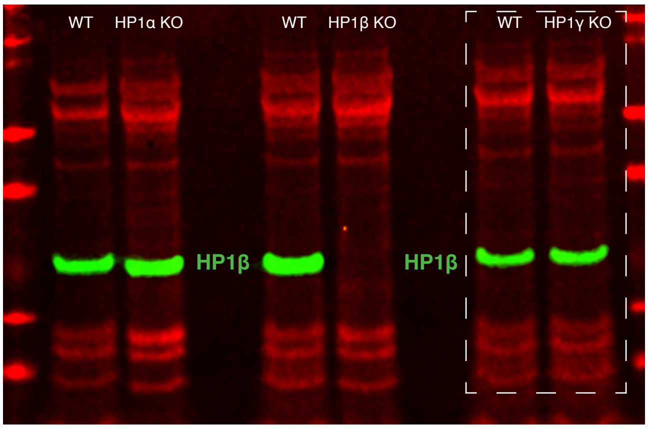

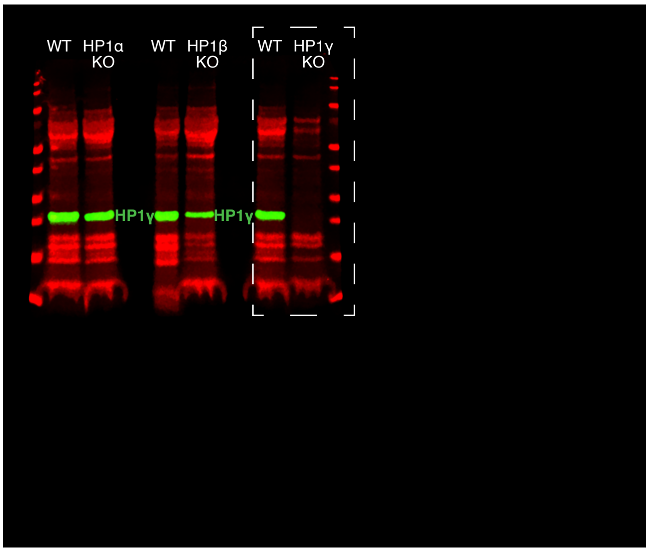

Source Data
