# Supplementary material for: Heterochromatic gene silencing controls CD4+ T cell susceptibility to regulatory T cell-mediated suppression in a murine allograft model
Source: Nat Commun. 2025 Jan 10;16:566. doi: 10.1038/s41467-025-55848-4 (PMC11723947; doi:10.1038/s41467-025-55848-4)
Supplement: Supplementary file 2 — Description of Additional Supplementary Files [file 41467_2025_55848_MOESM2_ESM.pdf]

Description of Additional Supplementary Files

File Name: Supplementary Data 1
Description: mRNA levels of the 54 genes identified in figure 3F, as measured by RNA–seq.

File Name: Supplementary Data 2
Description: mRNA levels of the 151 genes identified in figure 6E, as measured by RNA–seq.

File Name: Supplementary Data 3
Description: List of genes included in the 'Th1' gene set. The name of the gene and a reference are provided for each item.

File Name: Supplementary Data 4
Description: List of genes included in the 'Exhaustion' gene set. The name of the gene and a reference are provided for each item.
