# Supplementary material for: Heterochromatic gene silencing controls CD4+ T cell susceptibility to regulatory T cell-mediated suppression in a murine allograft model
Source: Nat Commun. 2025 Jan 10;16:566. doi: 10.1038/s41467-025-55848-4 (PMC11723947; doi:10.1038/s41467-025-55848-4)
Supplement: Supplementary file 7 — Reporting Summary [file 41467_2025_55848_MOESM7_ESM.pdf]

nature portfolio

Corresponding author(s): Olivier JOFFRE

Last updated by author(s): Nov 4, 2024

Reporting Summary

Nature Portfolio wishes to improve the reproducibility of the work that we publish. This form provides structure for consistency and transparency in reporting. For further information on Nature Portfolio policies, see our Editorial Policies and the Editorial Policy Checklist.

Statistics

For all statistical analyses, confirm that the following items are present in the figure legend, table legend, main text, or Methods section.

n/a	Confirmed	
☐	☒	The exact sample size (*n*) for each experimental group/condition, given as a discrete number and unit of measurement
☐	☒	A statement on whether measurements were taken from distinct samples or whether the same sample was measured repeatedly
☐	☒	The statistical test(s) used AND whether they are one- or two-sided<br>*Only common tests should be described solely by name; describe more complex techniques in the Methods section.*
☒	☐	A description of all covariates tested
☐	☒	A description of any assumptions or corrections, such as tests of normality and adjustment for multiple comparisons
☐	☒	A full description of the statistical parameters including central tendency (e.g. means) or other basic estimates (e.g. regression coefficient) AND variation (e.g. standard deviation) or associated estimates of uncertainty (e.g. confidence intervals)
☐	☒	For null hypothesis testing, the test statistic (e.g. *F*, *t*, *r*) with confidence intervals, effect sizes, degrees of freedom and *P* value noted<br>*Give P values as exact values whenever suitable.*
☒	☐	For Bayesian analysis, information on the choice of priors and Markov chain Monte Carlo settings
☐	☒	For hierarchical and complex designs, identification of the appropriate level for tests and full reporting of outcomes
☒	☐	Estimates of effect sizes (e.g. Cohen's *d*, Pearson's *r*), indicating how they were calculated

Our web collection on statistics for biologists contains articles on many of the points above.

Software and code

Policy information about availability of computer code

Data collection

Acquisition of flow cytometry data:
- BDFACSDiva (https://www.bdbiosciences.com/en-fr/products/software/instrument-software/bd-facsdiva-software)
- MACSQuantify™ Software (https://www.miltenyibiotec.com/FR-en/products/macsquantify-software.html#130-094-559)

Western blotting:
- Image Studio 5.2 (https://www.licor.com/bio/image-studio/)

NGS data:
All the NGS data were generated using the NovaSeq6000 sequencer. Assembly and annotation of the Mus musculus genomed were collected from the Ensembl database, v.107. mm10 blacklist were collected and converted to the mm39 genome with LiftOver tool from UCSC (https://genome.ucsc.edu/cgi-bin/hgLiftOver). To retrieve data from gene ontology and KEGG database, the R packages go.db v.3.18.0 and keggrest v.1.42.0 were used. org.mm.eg.db=3.18.0 was used to convert Ensembl ID to EntrezID.

Data analysis

- GrahPad Prism 10 Software (https://www.graphpad.com/scientific-software/prism/)
- FlowJo 10 Software (https://www.flowjo.com/solutions/flowjo/downloads)
- Microsoft Excel 16
- Image Studio Lite 5.2.5 (https://www.licor.com/bio/image-studio/)
- nf-core/RNA-seq pipeline v.3.9 with Nextflow v.21.10.6
- FastQC v.0.11.7 and Qualimap v.2.2.2
- Cutadapt v.3.4 with the wrapper Trim Galore! v.0.6.7

- STAR v.2.7.10a (for RNA-seq)
- BWA-MEM 2 v.2.2 (for ATAC-seq)
- samtools v.1.9
- MarkDuplicates from the Picard tools v.2.20.7
- HMMRATAC v.1.2.10 (ATAC-seq)
- BEDTools 2 v.2.29.0
- annotatePeaks.pl from HOMER v.4.10.4 (ATAC-seq)
- Salmon v.1.5.2 (RNA-seq)
- featureCounts from subread v.2.0.3 (ATAC-seq)
- R v.4.3
- DESeq2 v.1.38.3 (RNA-seq)
- edgeR v.3.40.2 (ATAC-seq)
- Seurat v.5.0.1 (scRNA-seq)
- WGCNA v.1.72-1
- clusterProfiler v.4.6.2
- summarizedexperiment v.1.20.0
- ggplot2 v.3.5.1
- ggprism v.1.0.4
- pheatmap v.1.0.12
- stringr v.1.5.1
- tidyr v.1.3.1 and dplyr v.1.1.3

Code used to perform the bioinformatics analyses is available through our Code Ocean capsules 4177274 [https://codeocean.com/capsule/6094357/tree/v2] and 5889808 [https://codeocean.com/capsule/6326593/tree/v1].

For manuscripts utilizing custom algorithms or software that are central to the research but not yet described in published literature, software must be made available to editors and reviewers. We strongly encourage code deposition in a community repository (e.g. GitHub). See the Nature Portfolio guidelines for submitting code & software for further information.

Data

Policy information about availability of data

All manuscripts must include a data availability statement. This statement should provide the following information, where applicable:

- Accession codes, unique identifiers, or web links for publicly available datasets
- A description of any restrictions on data availability
- For clinical datasets or third party data, please ensure that the statement adheres to our policy

Raw and processed data files from ATAC-seq and RNA-seq experiments have been deposited in the NCBI Gene Expression Omnibus (http://www.ncbi.nlm.nih.gov/geo/) under accession number GEO: GSE246831. The scRNAseq dataset used in this study has initially been published by Sade-Feldman and collaborators (Sade-Feldman M et al., Cell 2018). It is available in the NCBI Gene Omnibus Expression database under the accession number GEO: GSE120575. Code used to perform the bioinformatics analyses is available through our Code Ocean capsule 4177274 and 5889808.

Research involving human participants, their data, or biological material

Policy information about studies with human participants or human data. See also policy information about sex, gender (identity/presentation), and sexual orientation and race, ethnicity and racism.

Reporting on sex and gender	*Use the terms sex (biological attribute) and gender (shaped by social and cultural circumstances) carefully in order to avoid confusing both terms. Indicate if findings apply to only one sex or gender; describe whether sex and gender were considered in study design; whether sex and/or gender was determined based on self-reporting or assigned and methods used. Provide in the source data disaggregated sex and gender data, where this information has been collected, and if consent has been obtained for sharing of individual-level data; provide overall numbers in this Reporting Summary. Please state if this information has not been collected. Report sex- and gender-based analyses where performed, justify reasons for lack of sex- and gender-based analysis.*
Reporting on race, ethnicity, or other socially relevant groupings	*Please specify the socially constructed or socially relevant categorization variable(s) used in your manuscript and explain why they were used. Please note that such variables should not be used as proxies for other socially constructed/relevant variables (for example, race or ethnicity should not be used as a proxy for socioeconomic status). Provide clear definitions of the relevant terms used, how they were provided (by the participants/respondents, the researchers, or third parties), and the method(s) used to classify people into the different categories (e.g. self-report, census or administrative data, social media data, etc.) Please provide details about how you controlled for confounding variables in your analyses.*
Population characteristics	*Describe the covariate-relevant population characteristics of the human research participants (e.g. age, genotypic information, past and current diagnosis and treatment categories). If you filled out the behavioural & social sciences study design questions and have nothing to add here, write "See above."*
Recruitment	*Describe how participants were recruited. Outline any potential self-selection bias or other biases that may be present and how these are likely to impact results.*
Ethics oversight	*Identify the organization(s) that approved the study protocol.*

Note that full information on the approval of the study protocol must also be provided in the manuscript.

Field-specific reporting

Please select the one below that is the best fit for your research. If you are not sure, read the appropriate sections before making your selection.

[x] Life sciences   [ ] Behavioural & social sciences   [ ] Ecological, evolutionary & environmental sciences

For a reference copy of the document with all sections, see nature.com/documents/nr-reporting-summary-flat.pdf

Life sciences study design

All studies must disclose on these points even when the disclosure is negative.

Sample size	No sample-size calculation was performed. Independent experiments were performed at least three times to allow statistical analysis (with the exception of the xGvHD experiments which were carried out only twice) .
Data exclusions	No data exclusions was performed except when
Replication	To verify the reproducibility experimental findings were replicated several times.
Randomization	For in vivo studies, mice injected with either control or mutant naive CD4+ T cells were randomly distributed into cages.
Blinding	All experiments were performed without blinding.

Reporting for specific materials, systems and methods

We require information from authors about some types of materials, experimental systems and methods used in many studies. Here, indicate whether each material, system or method listed is relevant to your study. If you are not sure if a list item applies to your research, read the appropriate section before selecting a response.

Materials & experimental systems

n/a	Involved in the study	
[ ]	[x]	Antibodies
[x]	[ ]	Eukaryotic cell lines
[x]	[ ]	Palaeontology and archaeology
[ ]	[x]	Animals and other organisms
[ ]	[x]	Clinical data
[x]	[ ]	Dual use research of concern
[x]	[ ]	Plants

Methods

n/a	Involved in the study	
[x]	[ ]	ChIP-seq
[ ]	[x]	Flow cytometry
[x]	[ ]	MRI-based neuroimaging

Antibodies

Antibodies used

Flow cytometry:
AF700-labeled anti-CD45RA (HI100, BioLegend)
APC-conjugated anti-GM-CSF antibodies (MP1-22E9, BD Biosciences
APC-conjugated anti-Thy1.1 (OX-7, BD Biosciences)
APC-coupled anti-Foxp3 (236A/E7, Thermo Fisher Scientific)
APC-coupled anti-CD62L (MEL-14, Thermo Fisher Scientific)
APC-Cy7-conjugated anti-CD8 (eBioH35-17.2, Thermo Fisher Scientific)
APC-Cy7-coupled anti-CD45.1 (A20, BD Biosciences)
APC-Cy7-conjugated anti-CD3 (OKT3, BioLegend)
APC-eFluor-conjugated anti-Ly6G (RB6-8C5, Thermo Fisher Scientific)
APC-R700-coupled anti-IL-2 (JES6-5H4, BD Biosciences)
BB700-associated anti-TCR V6   (RR4-7, Thermo Fisher Scientific).
BB700-conjugated anti-IL-10 (JES3-19F, BD Biosciences)
BUV395-coupled anti-CD4 (SK3, BD Biosciences)
BUV605-conjugated anti-HLA-B7 (BB7.1, BD Biosciences)
BV421-coupled anti-TCR   (H57-597, BD Biosciences)
BV421-coupled anti-CD45.2 (104, BD Biosciences)
BV605-labeled anti-IL-17A (TC11-18H10.1, Biolegend)
BV605-conjugated anti-CD73 (TY/11.8, BioLegend)
BV711-coupled anti-FR4 (12A5, BD Biosciences)
BV750-associated anti-IL-2 (MQ1-17H12, BD Biosciences)

BV750-conjugated anti-IFN- (XMG1.2, BD Biosciences)
eFluor660-conjugated anti-Foxp3 (FJK-16s, Thermo Fisher Scientific)
FITC-conjugated anti-IFN- (XMG1.2, BD Biosciences)
FITC-coupled anti-Foxp3 (FJK-16s, Thermo Fisher Scientific)
FITC-conjugated anti-TNF (MP6 XT22, BD Biosciences)
FITC-conjugated anti-H-2Kb (AF6-88.5, BD Biosciences)
FITC-conjugated anti-TNF (MP6 XT22, BD Biosciences)
FITC-labeled anti-CD27 (LG.7F9, Thermo Fisher Scientific)
Pacific Blue-labeled anti-CD19 (1D3, BD Biosciences)
PE-conjugated anti-T-Bet (4B10, Thermo Fisher Scientific)
PE-coupled anti-RORt (Q31-378, BD Biosciences)
PE-coupled anti-IL-2 (JES6-5H4, Thermo Fisher Scientific)
PE-conjugated anti-CD25 (PC61.5 Thermo Fisher Scientific)
PE-coupled anti-LAG3 (C9B7W, BD Bioscience)
PE-coupled anti-H-2Kd (SF1-1.1, BD Biosciences)
PE-labeled anti-Granzyme B (GB11, BD Biosciences)
PE-CF594-coupled anti-CD69 (H1.2F3, BD Biosciences)
PE-Cy7-conjugated anti-IL-17A (eBio17B7, Thermo Fisher Scientific)
PE-Cy7-conjugated anti-PD-1 (J43, Thermo Fisher Scientific)
PE-Cy7-conjugated anti-CD44 (IM7, BD Biosciences)
PE-Cy7-conjugated anti-Foxp3 (FJK-16s, Thermo Fisher Scientific)
PerCP-Cy5.5-coupled anti-CD45.2 (104, BD Biosciences)
V500-labeled anti-CD4 (RM4-5, BD Biosciences)
rat IgG and anti-CD16/32 antibody (2.4G2, purified from B cell hybridoma supernatant)

Cell isolation:
APC-conjugated anti-Thy1.1 (OX-7, BD Biosciences)

Cell culture:
anti-CD3 (145-2C11, BioXcell)
anti-CD28 (37.51, BioXcell).
anti-IL-4 (11B11, BioXcell)
anti-IFN- (XMG1.2, BioXcell)

Western blotting
anti-HP1a (#2616, Cell Signaling)
anti-HP1b (#8676, Cell Signaling)
anti-HP1g (2MOD-1G6, purified from B cell hybridoma supernatant)

Validation

All antibody clones have been validated by the manufacturer (please refer to the data sheet for details).
Antibodies used in flow cytometry experiments are systematically titrated before use to ensure that staining is carried out under saturating conditions. Staining specificity has been assessed using Fluorescence Minus One (FMO) and isotype controls.

Animals and other research organisms

Policy information about studies involving animals; ARRIVE guidelines recommended for reporting animal research, and Sex and Gender in Research

Laboratory animals

The mutant mouse strains defective for HP1a, HP1b or HP1g were established at the Mouse Clinical Institute (IGBMC, Strasbourg, France). Mice homozygous for a conditional ready Hp1b (Hp1bflox) or Hp1g (Hp1gflox) allele and carrying the Cd4-cre transgene were crossed with mice only homozygous for the conditional ready allele to generate control and conditional HP1b-deficient (Hp1b-/-) or HP1g-deficient (Hp1g-/-) mice within the same litter. Mice carrying a conditional ready Hp1a (Hp1aflox) allele were crossed with CMV-cre transgenic mice to excise the floxed sequence. Mice heterozygous for the mutated allele (Hp1a+/−) were then systematically intercrossed to generate control (Hp1a+/+) and HP1a-deficient (Hp1a-/-) mice within the same litter. SUV39H1-deficient mice were kindly provided by T. Jenuwein (Max Planck Institute, Freiburg, Germany). As the gene encoding Suv39h1 is carried on the X chromosome, Suv39h1Y/+ males were crossed with heterozygous Suv39h1+/- females to obtain both Suv39h1Y/+ and Suv39h1Y/- males within the same litter. These different lines were generated and maintained on a mixed (129/Sv x C57BL/6) genetic background.
B6 Foxp3-Thy1.1 mice were provided by A Rudensky (Howard Hughes Medical Institute, Seattle).
All mutant mouse strains as well as C57BL/6J congenic mice expressing CD45.1 were bred under SOPF conditions at the Regional Center for Functional Exploration and Experimental Resources (CREFRE, INSERM UMS006, Toulouse).
C57BL/6J mice and B6D2F1 hybrids were obtained from Janvier Labs.
Sex-matched 6- to 12-weeks-old wild-type and mutant littermates were used and compared in all experiments.

Wild animals

The study did not involve wild animals.

Reporting on sex

To avoid potential problems linked to X chromosome inactivation in our NGS analyses, we worked with male mice.

Field-collected samples

The study did not involve samples collected from field

Ethics oversight	All experiments involving animals were conducted according to animal study protocols approved by the local ethics committee (#16-U1043-JVM-496, 21-U1291-JVM-292, PI-U1043-JVM-20 and #32669-2021080215068127).

Note that full information on the approval of the study protocol must also be provided in the manuscript.

Clinical data

Policy information about clinical studies

All manuscripts should comply with the ICMJEguidelines for publication of clinical research and a completedCONSORT checklist must be included with all submissions.

Clinical trial registration	Please, refer to Sade-Feldman M et al., Cell 2018 (https://doi.org/10.1016/j.cell.2018.10.038) for further details
Study protocol	Please, refer to Sade-Feldman M et al., Cell 2018 (https://doi.org/10.1016/j.cell.2018.10.038) for further details
Data collection	Please, refer to Sade-Feldman M et al., Cell 2018 (https://doi.org/10.1016/j.cell.2018.10.038) for further details
Outcomes	Please, refer to Sade-Feldman M et al., Cell 2018 (https://doi.org/10.1016/j.cell.2018.10.038) for further details

Plants

Seed stocks	*Report on the source of all seed stocks or other plant material used. If applicable, state the seed stock centre and catalogue number. If plant specimens were collected from the field, describe the collection location, date and sampling procedures.*
Novel plant genotypes	*Describe the methods by which all novel plant genotypes were produced. This includes those generated by transgenic approaches, gene editing, chemical/radiation-based mutagenesis and hybridization. For transgenic lines, describe the transformation method, the number of independent lines analyzed and the generation upon which experiments were performed. For gene-edited lines, describe the editor used, the endogenous sequence targeted for editing, the targeting guide RNA sequence (if applicable) and how the editor was applied.*
Authentication	*Describe any authentication procedures for each seed stock used or novel genotype generated. Describe any experiments used to assess the effect of a mutation and, where applicable, how potential secondary effects (e.g. second site T-DNA insertions, mosiacism, off-target gene editing) were examined.*

Flow Cytometry

Plots

Confirm that:

- [x] The axis labels state the marker and fluorochrome used (e.g. CD4-FITC).
- [x] The axis scales are clearly visible. Include numbers along axes only for bottom left plot of group (a 'group' is an analysis of identical markers).
- [x] All plots are contour plots with outliers or pseudocolor plots.
- [x] A numerical value for number of cells or percentage (with statistics) is provided.

Methodology

Sample preparation	Blood samples were treated with ACK (to deplete red blood cells).<br>Spleens were digested with Liberase and DNase.
Instrument	MACSQuant Analyzer 10 (Miltenyi Biotec)<br>LSRII (BD Biosciences)<br>Symphony A5 (BD Biosciences)<br>FACSAria Fusion cell sorter (BD Biosciences)
Software	BDFACSDiva (https://www.bdbiosciences.com/en-fr/products/software/instrument-software/bd-facsdiva-software)<br>FlowJo 10 Software (https://www.flowjo.com/solutions/flowjo/downloads)
Cell population abundance	Mouse Treg were isolated from the spleen of B6 Foxp3-Thy1.1 mice. For the in vitro experiments, Treg were enriched by positive selection on MS column (Miltenyi Biotec) following staining with APC-conjugated anti-Thy1.1 antibody (OX-7, BD Biosciences) and incubation with anti-APC microbeads (Miltenyi Biotec). The resulting cell suspension was systematically enriched to over 95% in CD4+Thy1.1+ cells (as determined by flow cytometry). For the in vivo experiments, the cell suspension underwent an additional selection step before culture. Treg, defined as CD4+CD25+Thy1.1+, were purified by fluorescence-activated cell sorting on a FACSAria Fusion cell sorter (BD Biosciences). The Treg population obtained was virtually pure after sorting and over 95% enriched after culture (as determined by flow cytometry).<br>Control and mutant mouse Tconv were enriched from the spleen of control and mutant littermates, respectively. Splenic naive CD4+ T cells were obtained by negative selection using the mouse naive CD4+ T Cell isolation kit (Miltenyi Biotec) according to the manufacturer's instructions. The CD4+CD62L+CD44-/low T cell population was routinely more than 95% pure (as determined by flow cytometry).

Human Treg were isolated from frozen PBMC using the EasySep Human CD4+CD127lowCD25+ Regulatory T Cell Isolation Kit (Stemcell) according to the manufacturer's instructions. The CD4+CD127lowCD25high Treg population was routinely more than 95% pure.
Human naive CD4+ T cells were isolated from frozen PBMC using the EasySep Human Naive CD4+ T Cell Isolation Kit II (Stemcell) according to the manufacturer's instructions. The CD3+CD4+CD45RA+ T cell population was routinely more than 95% pure (as determined by flow cytometry).

Gating strategy

Gating strategy used to analyze BM allograft rejection (Figures 1B-1C, 4A-4D, S4A-S4B)
FSC-A/SSC-A : all events except debris > FSH-H/FSC-W : single cells > SSC-H/SSC-W : single cells > H2Kb : live cells > H2Kd vs H2Kb

Gating strategy used to analyze the % of TCR VB6+ cells (Figure 2H)
FSC-A/SSC-A : all events except debris > FSH-H/FSC-W : single cells > SSC-H/SSC-W : single cells > FVD : live cells > H2Kb vs H2Kd : H2Kb+ cells > CD4vsThy1.1 : deletion injected Treg (Thy1.1+) > CD4 vs TCR Vb6 : CD4+TCRVb6+

Gating strategy used to analyze the % of anergic Tconv expressing PD-1, TIGIT or LAG-3 or secreting cytokines (Figures 5E-5H)
FSC-A/SSC-A : all events except debris > FSH-H/FSC-W : single cells > SSC-H/SSC-W : single cells > FVD-/H-2Kd- : live H-2Kd- cells > CD4+/Thy1.1- to eliminate Treg > CD4+/Vb6+ > CD73/FR4 double positive (anergic) vs other cells (non-anergic) > analysis of PD1+, TIGIT+, LAG3+, IFNg+, TNF+ or IL-2+ among anergic or non-anergic cells.

Gating strategy used to analyze human T cell ex vivo (Figures 7D-7J)
FSC-A/SSC-A : all events except debris > FSH-H/FSC-W : single cells > SSC-H/SSC-W : single cells > FVD : live cells > hCD3+/hCD4+ to gate on human T cell > hCD4+/HLA-B7- to eliminate Treg > CD45RA-/CD27- to gate effector memory cells > analysis of IFNg+, GM-CSF+, IL-10+, Granzyme B+, Foxp3+ and Tbet among total effector memory cells.

☒ Tick this box to confirm that a figure exemplifying the gating strategy is provided in the Supplementary Information.
